# Application of thoracic endovascular dissecting aneurysm repair for secondary type B aortic dissection

**DOI:** 10.5830/CVJA-2015-067

**Published:** 2015

**Authors:** Oguz Karahan, Orhan Tezcan, Demirtas Sinan, Ahmet Caliskan, Celal Yavuz

**Affiliations:** Medical School of Dicle University, Department of Cardiovascular Surgery, Diyarbakir, Turkey; Medical School of Dicle University, Department of Cardiovascular Surgery, Diyarbakir, Turkey; Medical School of Dicle University, Department of Cardiovascular Surgery, Diyarbakir, Turkey; Medical School of Dicle University, Department of Cardiovascular Surgery, Diyarbakir, Turkey; Medical School of Dicle University, Department of Cardiovascular Surgery, Diyarbakir, Turkey

**Keywords:** type A aortic dissection, surgery, endovascular intervention, hybrid procedure

## Abstract

Type A aortic dissection is an emergency condition that requires immediate surgery. Graft replacement of the ascending aorta is the main treatment for this disorder. However, after ascending aortic replacement, the dissection flap may progress to the distal side (to the descending aorta) and a new intimal tear may develop. In this study, we report on a 66-year-old woman who had a history of ascending aortic replacement six months earlier. She was admitted to hospital with a new onset of back pain. Computed tomography revealed a new dissection tear originating from the distal side of the subclavian artery orifice. Thoracic endovascular dissecting aneurysm repair (TEVDAR) was carried out on the patient. Additional complications were not observed in the postoperative period. Complete cure was provided and the patient was discharged on the fourth day after the operation. TEVDAR may be safe and effective in preventing progression of the aortic flap and the formation of a new intimal tear in type A aortic dissections. Optional hybrid interventions could ameliorate the outcomes in aortic dissection cases.

## Abstract

Aortic dissection (AD) is a life-threatening emergency situation that progresses rapidly. Early mortality rates are as high as 50%, even under optimal treatment conditions.^1^-^3^ Alternate treatment approaches may be used according to the specific AD subtype.^2^

The standard AD classification system used in clinical practice is Stanford’s classification. This system categorises AD into two classes, type A and B, according to the presence or absence of ascending aortic involvement.

Surgical replacement of the ascending aorta is indicated as the most appropriate curative therapy for Stanford type A AD. However, type B AD may initially be treated medically, with subsequent surgery or endovascular intervention.^2^,^3^ During the postoperative period, close monitoring of the progression of the flap, organ perfusion and other systemic events is critical. A rigorous postoperative follow up is required if the dissection flap involves the abdominal aorta or if the dissection has progressed significantly.^4^

Failure to closely monitor the disease progression in patients with type A AD undergoing surgical replacement of the aorta can result in significant clinical complications, such as secondary type B AD, as presented in the current case. The use of supplementary medication or hybrid interventions may improve the success rate of the initial ascending aortic graft replacement surgery.

Here, we report on a secondary type B AD patient who had previously been operated on for a type A AD. Thoracic aneurysm repair with endovascular graft is usually an elective procedure, but a dissecting aneurysm of the thoracic aorta is a more progressive and serious condition. We therefore undertook thoracic endovascular dissecting aneurysm repair (TEVDAR) instead of thoracic endovascular aneurysm repair (TEVAR). The presentation, management and clinical outcomes of the case are presented in the context of the current clinical literature.

## Case report

**Fig. 1.**
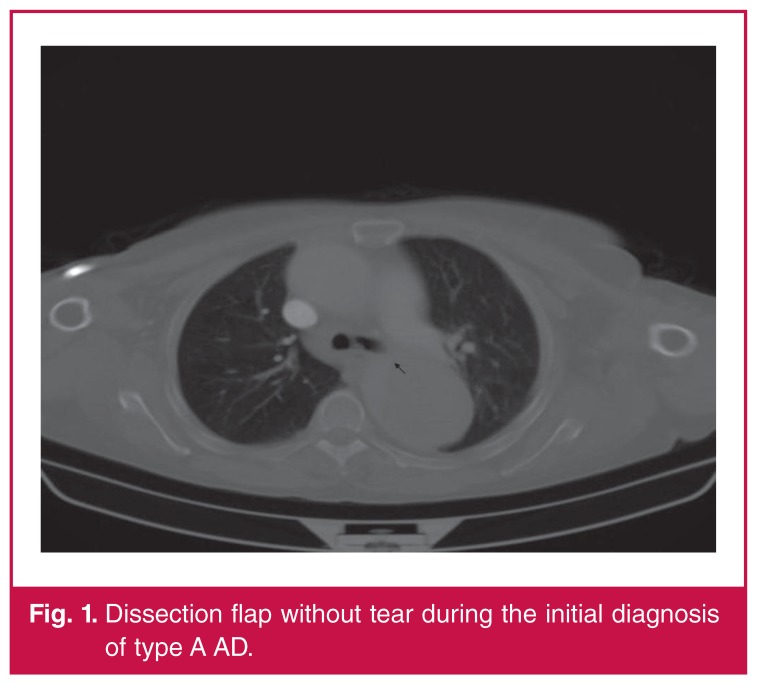
Dissection flap without tear during the initial diagnosis of type A AD.

A 66-year-old woman was admitted to hospital with severe backache. This patient had undergone ascending aortic replacement surgery to treat type A AD six months prior to the presentation (Fig. 1). The medical history of the patient included hypertension for the past 25 years, nephrectomy due to nephrolithiasis eight years earlier, polio sequela and a motor deficit of the left leg.

**Fig. 2.**
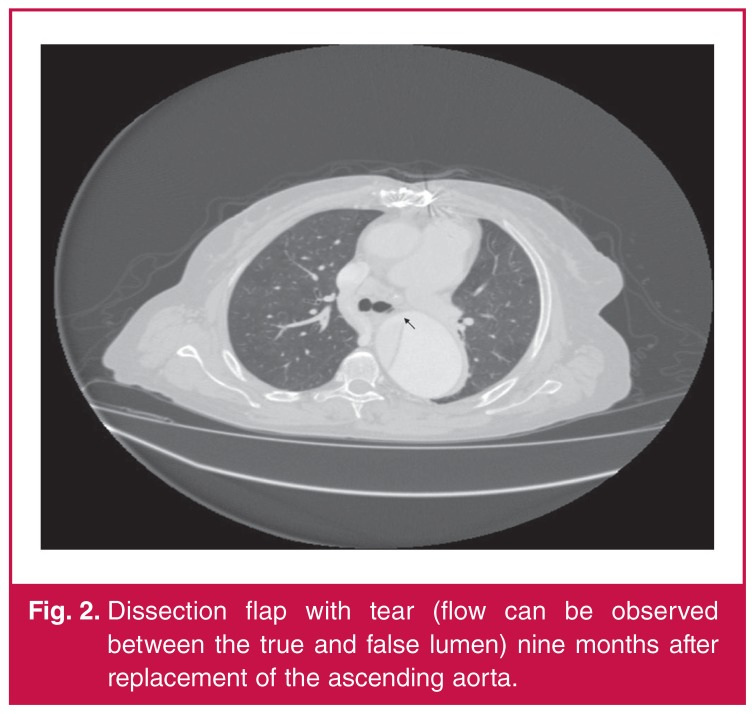
Dissection flap with tear (flow can be observed between the true and false lumen) nine months after replacement of the ascending aorta.

Her systolic blood pressure was 130 mmHg on the right arm and 110 mmHg on the left arm. All arterial pulses were determined by manual examination. Contrast-enhanced computed tomography revealed a type B dissection flap involving the left subclavian artery with retrograde progression. The diameter of the true lumen had narrowed significantly to < 10 mm, and the total diameter (with false lumen) was 43.7 mm at the widest section (Fig. 2). The peak aortic diameter was measured at 67.2 mm. We therefore initiated preparation for the TEVDAR surgery.

The patient underwent surgery under general anaesthesia. During the operation, an initial exploration of the right common femoral artery was conducted. A 5-F pigtail catheter was inserted into the ascending aorta through the right brachial artery. This artery was chosen to facilitate proximal imaging, as delivery of the catheter through the left brachial artery could have been inhibited by the presence of the thoracic aortic stent placed six months previously.

**Fig. 3.**
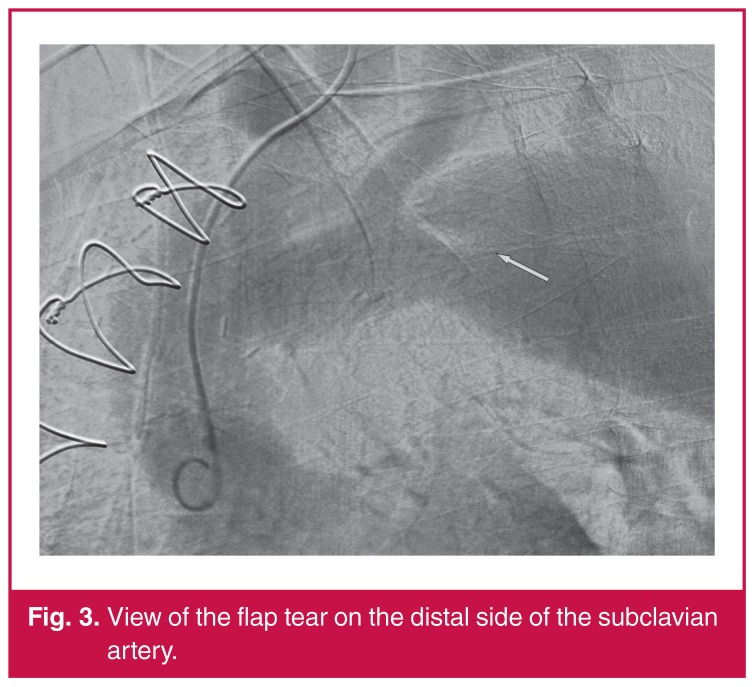
View of the flap tear on the distal side of the subclavian artery.

Contrast imaging of the aortic arch revealed the brachiocephalic truncus sourced from the left common carotid artery (Fig. 3) (bovine arch). Moreover, the origin of the retrograde dissection flap was identified 1 cm distal to the left subclavian artery in the contrast view (Fig. 3).

**Fig. 4.**
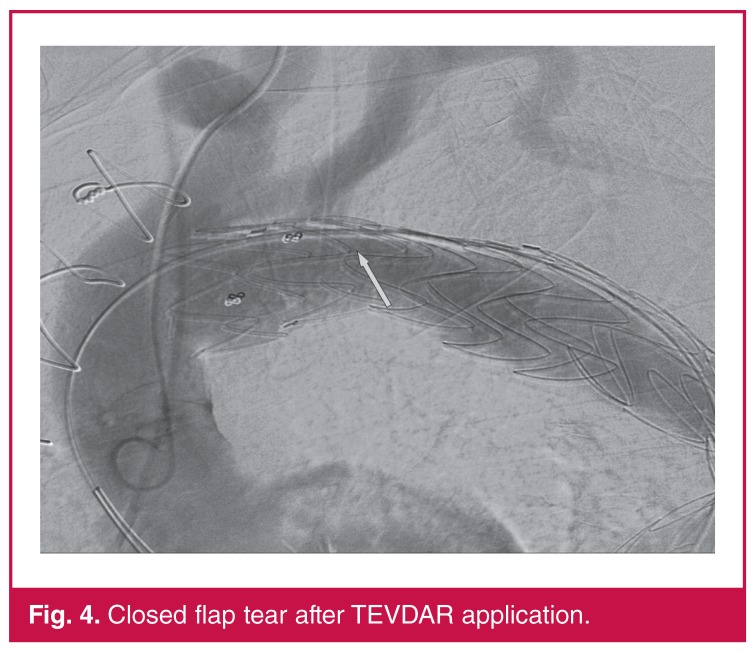
Closed flap tear after TEVDAR application.

Following the completion of the measurements, a 40 × 212-mm tube stent–graft was implanted into the descending aorta, including the proximal subclavian section. The placement of the tube stent–graft was challenging because of the narrowing of the true lumen and the high-angled aortic progression. The graft was placed using forced external manoeuvres. An extension tube stent–graft with a diameter of 42 × 112 mm was placed through the right common femoral artery. The correct placement of the extension tube stent–graft was confirmed with angiography and the application was concluded (Fig. 4). Primary repair of the right common femoral artery was conducted.

After surgery, no pulse deficit was observed in the left limb. The patient recovered in the intensive care unit and hydration was administered for deficient blood urine nitrogen and creatinine levels (due to the patient’s nephrectomy history). She was discharged on the fourth day after surgery.

## Discussion

All current treatment strategies for AD are associated with a high mortality rate. This risk is further increased by the extended patient transfer times. However, recent advances in surgical procedures may improve the overall morbidity and mortality rates in AD.^2^,^3^,^5^ In the present case, a brachiocephalic truncus in the left common carotid artery (bovine arch) was detected by contrast-enhanced computed tomography.

Hornick *et al.* retrospectively analysed a series of AD cases and they concluded that bovine arch anatomy is not associated with increased incidence of AD compared with the normal anatomical configuration. Therefore bovine arch anatomy was likely not a significant factor in the development of AD in the present case.^6^

The primary treatment strategy for type A AD is graft replacement of the ascending aorta.^5^,^7^ However, dissection or distension of the distal aorta may be neglected in most cases. In the present case, the patient had undergone replacement of the ascending aorta due to type A dissection nine months earlier.

Upon presentation, contrast-enhanced computed tomography (CT) detected a progression of the previous flap to the level of the renal artery. No additional progression of the dissection flap had been observed in the previous CT images (Fig. 3)). In the earlier CT examination, the true lumen had retained the contrast. However, our examination nine months later showed the contrast agent passing through the false lumen.

Despite the absence of a rupture in the aorta, these recent flap changes could have caused organ ischaemia due to the narrowing of the lumen diameter. Therefore, we proposed that the type B dissection was closely related to the previous type A dissection in this case. Moreover, the same risk factors that resulted in the previous dissection of the ascending aorta (hypertension, etc) could also have resulted in disruption of the distal section of the aortic intima, even though the damage to the proximal aorta had been completely repaired.

## Conclusion

TEVDAR application is beneficial to most patients diagnosed with type B AD. Although this procedure is associated with higher costs, the benefits of this intervention include reduced risk of complications, shorter recovery time in the intensive care unit and a more rapid return to normal quality of life. Additionally, the management of type A AD using surgical and medical hybrid therapy may be critical to the prevention of secondary complications, such as the development of a secondary type B AD. In high-risk AD cases, TEVDAR may result in improved outcomes and a better quality of life.
